# Interactive Effects of Sertraline and Diphenhydramine on Biochemical and Behavioral Responses in Crucian Carp (*Carassius auratus*)

**DOI:** 10.3390/ijerph16173137

**Published:** 2019-08-28

**Authors:** Zhengxin Xie, Guanghua Lu

**Affiliations:** 1 School of Resources and Environment, Anhui Agricultural University, Hefei 230036, China; 2 Water Conservancy Project & Civil Engineering College, Tibet Agriculture & Animal Husbandry University, Linzhi 860000, China; 3 College of Environment, Hohai University, Nanjing 210098, China

**Keywords:** psychiatric pharmaceutical, mixture, behavior, oxidative stress, neurotoxicity

## Abstract

The ecotoxicity of psychiatric pharmaceuticals to aquatic organisms is being increasingly recognized. However, current ecological studies focus on the effects of individual psychiatric pharmaceuticals, with little attention being given to their combined effects. In this study, the interactive effects of two psychiatric pharmaceuticals, sertraline (SER) and diphenhydramine (DPH), on bioconcentration and biochemical and behavioral responses were investigated in crucian carp (*Carassius auratus*) after seven days of exposure. DPH was found to increase the accumulation of SER in fish tissues relative to SER-alone exposure. In addition, the mixture of SER and DPH significantly changed the activities of antioxidant enzymes and led to significant increases in malondialdehyde content, relative to SER alone. Concerning the neurotoxicity, relative to SER-alone exposure, brain AChE activity was significantly enhanced in fish following the combined exposure. Regarding behavioral responses, swimming activity and shoaling behavior were significantly altered in co-exposure treatments compared with the SER alone. Moreover, the inhibition effects on the feeding rates were increased in co-exposure treatments compared to SER alone. Collectively, our results suggest that the mixtures of psychiatric pharmaceuticals may pose more severe ecological risks to aquatic organisms compared to these compounds individually.

## 1. Introduction

Pharmaceuticals have gained growing attention for their potential risks to aquatic ecosystems [1]. Due to their large usage and incomplete removal in sewage treatment plants (STPs) or the lack of STPs, pharmaceuticals have been widely found in aquatic environments [2,3,4,5]. As an important group of pharmaceuticals, psychiatric compounds have received great attention. These pharmaceuticals commonly include antidepressants, anxiolytics, sedatives, hypnotics, and antiepileptics. Because of their increasing usage and wide occurrence in aquatic environments globally, psychiatric pharmaceuticals have raised a growing concern for their negative effects on aquatic organisms [1]. Recent studies have demonstrated the toxicity of these pharmaceuticals to various aquatic organisms, even at environmentally relevant concentrations [6,7,8]. In particular, a growing body of studies have focused on the effects of psychiatric pharmaceuticals on the behavior of aquatic species, since these pharmaceuticals are designed to alter behavior [9]. The behavioral alterations are not lethal, but can indirectly influence entire populations, and thus ecosystem functioning [10].

In general, psychiatric pharmaceuticals exist as mixtures in aquatic environments [11,12,13,14]. As a result, aquatic organisms in their habitats may be exposed to the mixtures of psychiatric pharmaceuticals, rather than individual compounds. The pharmaceuticals in mixtures may interact through a combination of mechanisms, such as binding to a receptor, regulating gene expression, or changing the cellular metabolism [15]. Consequently, the interactions of psychiatric pharmaceuticals may alter their bioaccumulation and toxic effects in aquatic organisms in comparison with the effects of these compounds individually. However, most of the previous ecotoxicological studies only focus on influences of individual psychiatric pharmaceuticals. Clearly, information on the interaction of psychiatric pharmaceuticals in mixtures is an urgent need if we are to fully assess their risks to the aquatic ecosystem.

The present study was carried out to investigate possible adverse effects triggered by a combination of sertraline (SER) and diphenhydramine (DPH). SER is among the most widely prescribed selective serotonin reuptake inhibitors (SSRIs) [16]. DPH is a commonly used over-the-counter antihistamine. DPH reduces allergic response by competitively antagonizing histamine H1 receptor. Additionally, DPH can inhibit the reuptake of serotonin by the presynaptic membrane [17]. Due to their frequent usage, SER and DPH have been widely detected in surface water at maximum concentrations of 0.49 μg·L^−1^ and 1.4 μg·L^−1^, respectively [13,18]. It has been previously reported that the acute mortality and reproductive effects induced by the mixture of SER and DPH were additive in *Ceriodaphnia dubia* [19]. This observation suggests that the mixture of SER and DPH may pose stronger impacts on aquatic organisms. However, standard acute toxicity tests may not be sensitive enough to assess the effects of pharmaceuticals on aquatic organisms [20]. It is necessary to use more sensitive endpoints in the assessment of the risk of pharmaceuticals in aquatic environments. In addition, the combined effects of SER and DPH on aquatic organisms at higher trophic levels, such as fish, are unknown. Our previous studies showed that SER and DPH accumulated in crucian carp (*Carassius auratus*) and induced significant changes in the acetylcholinesterase (AChE) and antioxidant enzyme activities, as well as behaviors at low exposure levels (4.36 μg·L^−1^ for SER and 4.23 μg·L^−1^ for DPH) [16,21]. Considering the additive toxicity observed in *Ceriodaphnia dubia*, it raises the question of whether the mixture of SER and DPH can change the bioaccumulation of these compounds and result in stronger behavioral and biochemical responses in fish species.

Therefore, the goal of this study was to: (1) investigate the bioconcentration and distribution of SER alone, and in combination with DPH in crucian carp; (2) assess the activity of AChE in the brain and responses of antioxidative defense systems (superoxide dismutase (SOD), catalase (CAT), glutathione peroxidase (GPx), glutathione S-transferase (GST), and malondialdehyde (MDA)) in the liver of crucian carp exposed to SER alone and its combination with DPH; and (3) examine the effects of SER and its mixture with DPH on the swimming activity, shoaling behavior, and feeding rate of crucian carp.

## 2. Materials and Methods

### 2.1. Chemicals and Reagents

The analytical standard of SER and DPH were supplied by Sigma-Aldrich (Flanders, New Jersey, USA). The physicochemical properties of SER and DPH are listed in Table 1. Biochemical assay kits were provided by the Nanjing Jiancheng Bioengineering Institute. The HPLC grade of methanol and acetone were acquired from Merck Serono Co., Ltd. (Darmstadt, Germany).

### 2.2. Experimental Species and Exposure Conditions

Crucian carp (16.3 ± 1.54 g) were supplied by Nanjing Institute of Fishery Sciences (Nanjing, China). Fish were acclimatized to aquarium conditions (500 L) for two weeks. The culture medium was dechlorinated municipal water, and quality parameters were as follows: pH, 6.9 ± 0.1; dissolved oxygen, 6.8 ± 0.3 mg L^−1^; and total hardness, 121.6 ± 4.8 mg L^−1^ CaCO_3_. The fish were maintained at 20 ± 1 °C with a 12 h:12 h light–dark photoperiod cycle.

The fish were treated with SER alone and its combination with DPH for 7 days at the following concentrations: SER (5 μg·L^−1^, named as S) and SER +DPH (5 μg·L^−1^ + 1 μg·L^−1^, 5 μg·L^−1^ +5 μg·L^−1^, 5 μg·L^−1^ +25 μg·L^−1^ and 5 μg·L^−1^ +125 μg·L^−1^, named as S + D1, S + D2, S + D3, and S + D4, respectively). A dechlorinated water control treatment and solvent control treatment (0.1% methanol) were also set in the exposure treatments. Each treatment was prepared in six replicate tanks (30 L) with four fish per tank. Fish were not fed during the exposure period. The exposure solutions were refreshed every 24 h. After the exposure experiment, six fish were sampled in each treatment for biochemical assay and chemical analysis. The liver, brain, gills, and muscle were dissected, immediately frozen in liquid nitrogen, and stored at −80 °C until the assays. The remaining 18 fish in each treatment were used for behavioral trials at the end of exposure. In addition, water samples were collected daily to measure the exposure concentrations of SER and DPH.

### 2.3. Sample Extraction and Chemical Analysis

SER and DPH in water and tissue samples were extracted using the methods described in our previous studies [16,21]. The detection and quantification of target compounds were conducted using an Agilent 1290 ultra-high-performance liquid chromatograph (UPLC; Agilent, Waldbronn, Germany) coupled to an Agilent 6460 triple quadrupole mass spectrometer. Details of the extraction procedures and analyses are listed in the Supplementary Materials.

### 2.4. Enzyme Assays

The tissue sample was homogenized in chilled physiological saline solution (1:10, w/v). The homogenates were then centrifuged for 10 min (10,000 *g*) at 4 °C. The obtained supernatant was used to determine enzyme activity, MDA content, and protein concentration using bioassay kits according to the manufacturer’s protocol. Enzyme activity is reported as unit (U) mg^−1^ protein, and MDA content is expressed in nmol mg^−1^ protein.

### 2.5. Behavioral Tests

At the end of exposure, the swimming activity, shoaling behavior, and feeding rate were measured following the previous methods [21]. Swimming activity was defined as the number of lines crossed both parallelly and vertically. Shoaling behavior was assessed by the number of times the test fish crossed the parallel line and the duration of time it spent away from the shoal. Feeding rate was quantified as the latency for fish to capture the first, fifth, and last (10th) midge larvae. The detailed protocols of behavioral tests are provided in the Supplementary Materials.

### 2.6. Data Analyses

Shapiro-Wilk’s and Levene’s tests were used to check the normality and homogeneity of variances of data, respectively. One-way ANOVA (Dunnett’s test) was used to assess the differences of data from different treatments. There was considered to be a significant difference at *p* < 0.05. Statistical analyses were conducted with SPSS 17.0 (SPSS, Inc., Chicago, IL, USA).

## 3. Results and Discussion

### 3.1. Concentrations of SER and DPH in Exposure Solutions

The measured concentrations of SER and DPH in the exposure solutions are shown in Table 2. No SER and DPH was detected in water samples from the control groups. The actual treatment concentrations of SER and DPH in the exposure solutions were generally consistent with their correspondingly nominal concentrations (within ± 20%).

### 3.2. Bioconcentration of SER

**Figure 1:**
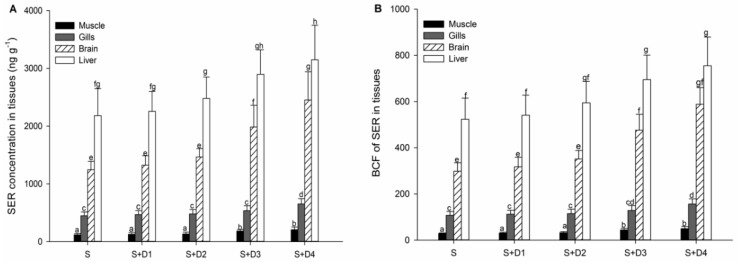
**(A)** Concentrations and **(B)** bioconcentration factors (BCFs) of SER in different fish tissues after seven days of exposure. Data are mean ± SD (*n* = 3). Different letters indicate significant differences (*p* < 0.05). S: 5 μg·L^−1^ SER, S + D1: 5 μg·L^−1^ SER + 1 μg·L^−1^ DPH, S + D2: 5 μg·L^−1^ SER + 5 μg·L^−1^ DPH, S + D3: 5 μg·L^−1^ SER + 25 μg·L^−1^ DPH, S + D4: 5 μg·L^−1^ SER + 125 μg·L^−1^ DPH.

Tissue concentrations of SER in fish over a seven-day exposure duration are shown in Figure 1A. Levels of SER were not detectable in fish tissues from the control treatment. In the treatment with SER alone, the highest concentrations were found in the liver (2183 ng·g^−1^), followed by the brain (1244 ng·g^−1^), gills (448 ng·g^−1^), and muscle (448 ng·g^−1^). Compared to SER-alone treatment, co-exposure of SER and DPH did not change the tissue distribution pattern of SER, but increases in the tissue concentrations of SER were found in all co-exposure treatments, reaching significance in the treatments of SER plus two high concentrations of DPH (S + D3 and S + D4). So far, few studies have investigated the interactions of pharmaceutical mixtures on their bioaccumulation in aquatic organisms. Franzellitti et al. [22] found that fluoxetine co-exposure facilitated the bioaccumulation of propranolol in marine mussels (*Mytilus galloprovincialis*). More recently, Ding et al. [23] reported that the addition of fluoxetine increased the accumulation of roxithromycin and propranolol in crucian carp (*Carassius auratus*). As suggested by these studies, the elevated tissue concentrations of roxithromycin and propranolol in mussels and fish might be due to drug–drug interactions resulting from the inhibition of fluoxetine on cytochrome P450 (CYP) activity, and thus of clearance, eventually increasing the accumulation of these pharmaceuticals. In humans and mammals, SER is mainly metabolized by the CY2D6 enzyme, while DPH is a strong inhibitor of the CY2D6 enzyme [24,25]. Thus, DPH might inhibit the CY2D6 enzyme activity in fish liver that subsequently promoted SER accumulation. However, whether fish contain the CYP2D6 enzyme is currently unclear. Therefore, to reveal the interaction mechanism of DPH and SER, further work is still required to measure CYP2D6 activity using the method of dextromethorphan oxidation in the liver microsomal fractions of crucian carp.

To assess the bioconcentration potential of SER, the bioconcentration factor (BCF) was defined as the ratio of SER concentration in fish tissue to that in water. The BCFs in the liver, brain, gills, and muscle of fish following exposure to SER alone were 523, 298, 107, and 28.3, respectively (Figure 1B). The observed BCFs in this study are comparable to those observed by Xie et al. [16] in crucian carp exposed to 4.36 μg·L^−1^ SER, which were 626 in the liver, 285 in the brain, 146 in the gills, and 46.8 in muscle. In comparison with SER-alone treatment, tissue BCFs in the co-exposure treatments of SER plus two high concentrations of DPH were significantly increased. The highest BCFs in co-exposure treatments were 755 in the liver, 588 in the brain, 157 in the gills, and 49.6 in muscle. These results suggested that DPH could facilitate the accumulation extent of SER in crucian carp.

### 3.3. Biochemical Effects

**Figure 2:**
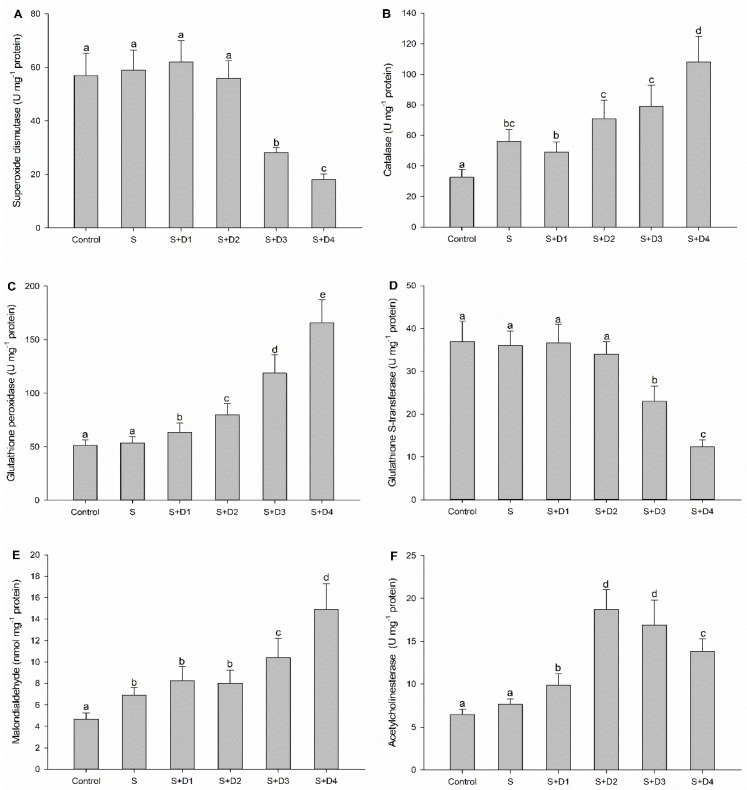
Biochemical responses in tissues of fish following various treatments with SER alone or its combination with DPH for seven days. Different letters indicate significant differences (*p* < 0.05). (**A**) superoxide dismutase, (**B**) catalase, (**C**) glutathione peroxidase, (**D**) glutathione S-transferase, (**E**) malondialdehyde, (**F**) acetylcholinesterase. S: 5 μg·L^−1^ SER, S + D1: 5 μg·L^−1^ SER + 1 μg·L^−1^ DPH, S + D2: 5 μg·L^−1^ SER + 5 μg·L^−1^ DPH, S + D3: 5 μg·L^−1^ SER + 25 μg·L^−1^ DPH, S + D4: 5 μg·L^−1^ SER + 125 μg·L^−1^ DPH.

It is known that many xenobiotics, such as pharmaceuticals, can induce the overproduction of reactive oxygen species (ROS), leading to lipid peroxidation (LPO) in aquatic organisms [26,27,28,29]. Antioxidant enzymes, including SOD, CAT, and GPx, are responsible for neutralizing the generation of ROS in cell metabolism. SOD has a main function in catalyzing the dismutation of the superoxide anion to H_2_O and H_2_O_2_, which is subsequently degraded by CAT to non-toxic H_2_O and O_2_ [30]. GPx can work together with CAT as scavengers of H_2_O_2_ and other hydroperoxides [31]. As an important phase II biotransformation enzyme, GST is also capable of neutralizing lipoperoxidation products through the oxidation of reduced glutathione [32]. In the present study, the activities of SOD, GPx, and GST were not significantly changed by SER alone compared to control treatment, whereas significant induction was observed for CAT activity (Figure 2A–D). Regarding mixture effects, SOD and GST were significantly inhibited by SER plus two high concentrations of DPH, whereas CAT and GPx were significantly induced in all co-exposure treatments. These results suggested the mixture of SER and DPH provoked stronger disturbance on the antioxidant system in fish liver compared to SER alone. To confirm whether oxidative stress occurred, LPO levels in fish liver were assessed by measuring MDA content (a by-product of LPO). As shown in Figure 2E, a significant increase was observed for MDA contents in fish liver from SER-alone treatment, indicating the occurrence of oxidative stress. Relative to the MDA content in the liver of fish upon exposure to SER alone, those in the co-exposure treatments of SER plus two high concentrations of DPH were significantly increased. These observations demonstrated that SER combined with high concentrations of DPH could induce severer oxidative damage in fish liver.

AChE hydrolyses the neurotransmitter acetylcholine (ACh) in cholinergic synapses. This enzyme plays an important role in neurological function, which is crucial to control the physiological and behavioral processes in many organisms [33]. Thus, AChE has been suggested as a suitable biomarker of neurotoxicity induced by many pollutants, such as pesticides, heavy metals, and pharmaceuticals [34,35,36]. In the present study, AChE activity was significantly increased in the brain of fish treated with SER alone. In addition, the induction of AChE activity was significantly increased by co-exposure treatments compared to SER alone (Figure 2F). These results were consistent with previous studies, where the brain AChE activity of crucian carp was significantly induced by SER at 4.36 μg·L^−1^ and DPH at 4.23 μg·L^−1^ [16,21]. Previous studies have shown that serotonin receptor activation may stimulate ACh release in different cell types [37,38,39]. Considering these previous findings, we hypothesized that SER and DPH may enhance the activity of brain serotonin receptors resulting in the increase of ACh, and then trigger a consequent induction of AChE activity. It has been well-established that increased AChE activity can be detected in apoptotic cells after apoptotic stimuli in vitro and in vivo [40]. In the face of apoptotic events in different types of cells resulting from SSRI exposure [41,42,43], SER and its mixture with DPH may promote apoptosis in brain cells of crucian carp. Moreover, the brain neurotransmitter level and enzyme function are also associated with behaviors [44]. Thus, our observations suggested that co-exposure of SER and DPH may lead to stronger adverse impacts on the physiological and behavioral processes of crucian carp.

### 3.4. Behavioral Effects

**Figure 3:**
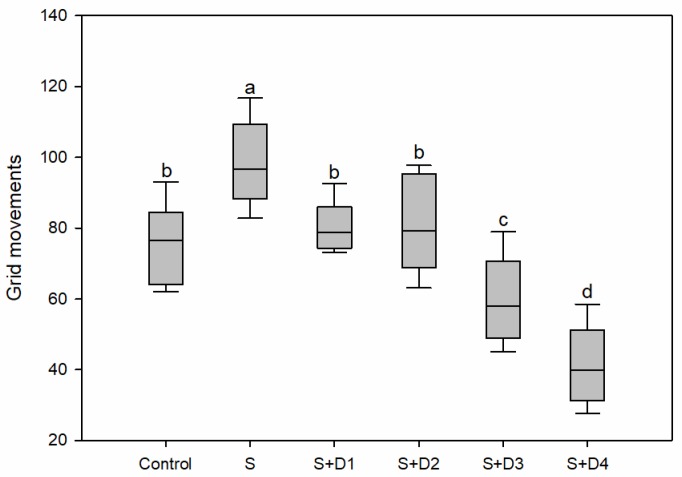
Swimming activity of fish following various treatments with SER alone or its combination with DPH for seven days. Swimming activity is measured as the number of grid lines crossed. Different letters indicate significant differences (*p* < 0.05). S: 5 μg·L^−1^ SER, S + D1: 5 μg·L^−1^ SER + 1 μg·L^−1^ DPH, S + D2: 5 μg·L^−1^ SER + 5 μg·L^−1^ DPH, S + D3: 5 μg·L^−1^ SER + 25 μg·L^−1^ DPH, S + D4: 5 μg·L^−1^ SER + 125 μg·L^−1^ DPH.

Swimming activity is of high ecological importance because it can be directly linked to the survival, growth, and reproduction of aquatic organisms [44]. In this study, the exposure to SER alone led to a significant increase of swimming activity. In contrast, significant decreases were observed in fish exposed to the co-exposure of SER and DPH at two high concentrations (Figure 3). These results suggested the presence of DPH seemed to comprise the induction effects caused by SER. The inhibition effect of DPH on the swimming activity of crucian carp was previously observed in our published work [21]. The discrepancy between SER and DPH may be attributed to their different chemical structure and mechanism of action. SER is a secondary amine, whereas DPH is a tertiary amine. Regarding the mechanism of action, SER elicits therapeutic effects through increasing serotonergic neurotransmission by blocking reuptake of serotonin by presynaptic serotonin reuptake transporters [16]. The primary mechanism of action for DPH is antagonism of the histamine H1 receptor. DPH can also inhibit serotonin reuptake at the synaptic cleft [17]. It is well-recognized that behavioral alterations could lead to adverse effects on the fitness and survival of the organism. The impairment of swimming activity could negatively affect feeding and growth, leading to a reduction in fitness [45]. On the other hand, excessively increased swimming activity may increase predation risk and result in reduced fitness [9].

**Figure 4:**
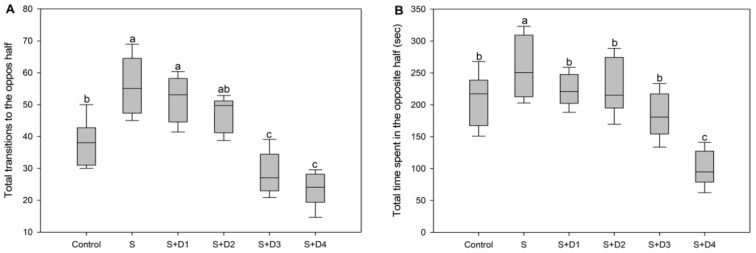
Shoaling behavior of fish following various treatments with SER alone or its combination with DPH for seven days. Shoaling is expressed as (**A**) total transitions to the opposite half and (**B**) total time spent in the opposite half. Different letters indicate significant differences (*p* < 0.05). S: 5 μg·L^−1^ SER, S + D1: 5 μg·L^−1^ SER + 1 μg·L^−1^ DPH, S + D2: 5 μg·L^−1^ SER + 5 μg·L^−1^ DPH, S + D3: 5 μg·L^−1^ SER + 25 μg·L^−1^ DPH, S + D4: 5 μg·L^−1^ SER + 125 μg·L^−1^ DPH.

As shown in Figure 4, significant inhibition of shoaling behavior was observed in SER-alone treatment. Shoaling behavior was also significantly decreased by the co-exposure of SER with the lowest concentration of DPH. However, shoaling behavior increased with an increasing concentration of DPH, and significant enhancements relative to the control level were observed in the treatments of SER plus DPH at the two high-exposure concentrations. Crucian carp is a social fish that typically maintains loose groups or shoals [46]. Shoaling behaviors offer benefits for social fish species at individual and population levels of organization by increasing survival and fitness [47]. Decreased shoaling behavior may result in reduction of fitness and survival, foraging, migration, spawning, and predator avoidance [48]. However, increased shoaling behavior may not always have positive effects on fish. Unnecessary shoaling behavior will decrease the time available for other essential activities, such as foraging and reproduction [44].

**Figure 5:**
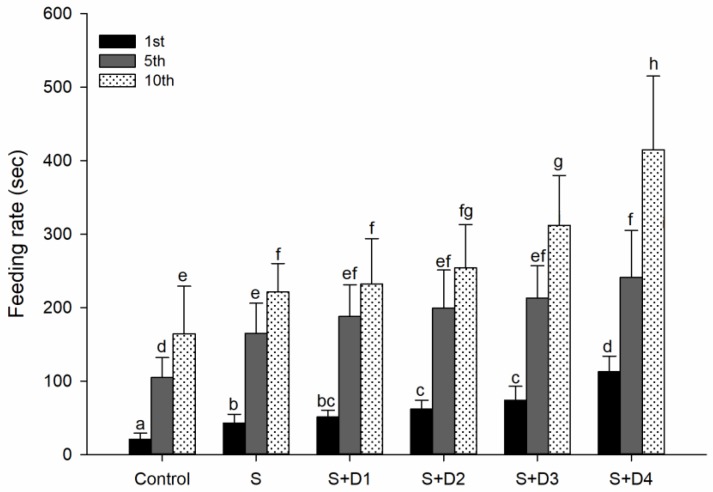
Feeding rate of fish following various treatments with SER alone or its combination with DPH for seven days. Feeding rate is assessed by the latency to capture the first, fifth and 10th midge larvae. Different letters indicate significant differences (*p* < 0.05). S: 5 μg·L^−1^ SER, S + D1: 5 μg·L^−1^ SER + 1 μg·L^−1^ DPH, S + D2: 5 μg·L^−1^ SER + 5 μg·L^−1^ DPH, S + D3: 5 μg·L^−1^ SER + 25 μg·L^−1^ DPH, S + D4: 5 μg·L^−1^ SER + 125 μg L^−1^ DPH..

To assess more direct ecological effects of SER and its mixture with DPH, feeding rate was measured as the latency for the fish to capture the first, fifth, and 10th midge larvae. As indicated by the increased latency for fish to capture midge larvae, SER-alone exposure significantly decreased the feeding rates of crucian carp. Compared to individual SER exposure, the addition of DPH increased the inhibition of feeding rate, reaching significant increases in the treatments of SER plus DPH at the two high concentrations (Figure 5). These results suggested that mixtures posed severer impairment of feeding performance relative to SER alone. Decreased feeding rate has also been previously reported in fish species and dragonflies upon exposure to other psychiatric pharmaceuticals, such as fluoxetine, citalopram, and tramadol [49,50]. Decreased feeding rates could negatively affect the growth, reproduction, and survival of individual fish [51]. In addition, the decreased feeding rate of fish may, over time, influence predator–prey interaction strength, leading to changes in population dynamics, community structure, and food-web stability [50,52]. Given the additive effects of SER and DPH on the feeding rate, our results suggested that ecological risks of psychiatric pharmaceuticals may be underestimated without considering the mixture effects.

In the present study, the exposure concentration of SER was much higher than the maximum concentration in surface water (0.49 μg·L^−1^) [13]. In addition, the significantly interactive effects of SER and DPH generally took place at DPH levels which were 18 and 89 times higher than the highest concentration found in surface water (1.4 μg·L^−1^) [18]. These results suggested that SER and DPH may not have interactive effects on wild fish in aquatic environments. However, it should be noted that our experiments were conducted in a short-term exposure period. In natural water, fish are likely to be exposed to xenobiotics for their entire life span. Thus, it is difficult to rule out the possibility that the interactive effects of SER and DPH will occur in fish exposed to low concentrations of these compounds in a long-term exposure period. Clearly, further research is required to evaluate the chronically interactive effects of SER and DPH at environmentally relevant concentrations.

### 3.5. Principal Component Analysis (PCA)

**Figure 6:**
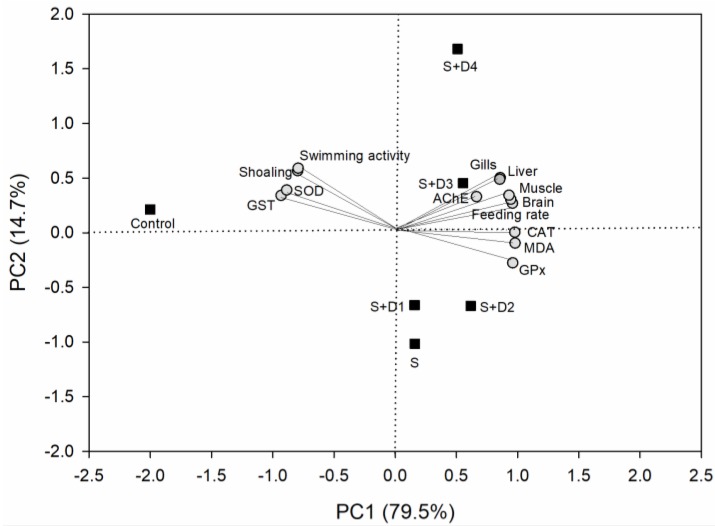
Principal component analysis (PCA) bi-plot of biochemical and behavioral endpoints and tissue concentrations of SER in fish under different treatments. S: 5 μg·L^−1^ SER, S + D1: 5 μg·L^−1^ SER + 1 μg·L^−1^ DPH, S + D2: 5 μg·L^−1^ SER + 5 μg·L^−1^ DPH, S + D3: 5 μg·L^−1^ SER + 25 μg·L^−1^ DPH, S + D4: 5 μg·L^−1^ SER + 125 μg·L^−1^ DPH.

PCA analysis was performed to interpret the relationships among the behavioral and biochemical endpoints and tissue concentrations of SER in crucian carp subjected to different treatments. As shown in Figure 6, the first (PC 1) and second principal component (PC 2) with eigenvalues greater than 1.0 represented 79.5% and 14.7% of the total variance, respectively. PCA provided some interesting information: (1) all treatments showed evident separation from the control treatment. (2) SER-alone treatment was separated from co-exposure treatments, especially for the SER plus two high concentrations of DPH, providing important hints for interactions of SER and DPH. (3) SOD, GST, swimming activity, and shoaling behavior were clustered, whereas other endpoints formed another group, due to their similar response patterns. Interestingly, SOD and GST were separated from CAT and GPx, although these enzymes all act against ROS. As mentioned in Section 3.3, these enzymes are responsible for the removal of different kinds of ROS. Thus, our observation may have been due to the differences in the functions of these enzymes. (4) Co-exposure treatments were correlated with tissue concentrations of SER, suggesting that the addition of DPH influenced the bioconcentration of SER. (5) AChE, CAT, GPx, and MDA content were associated with the feeding rate, indicating that neurotoxicity and oxidative stress induced by SER and its mixtures with DPH may promote behavioral alterations of fish.

## 4. Conclusions

The results of this study showed that the presence of DPH elevated the tissue concentrations of SER, indicating an increased bioconcentration risk of fish upon exposure to pharmaceutical mixtures. Antioxidant enzymes and MDA in fish were more sensitive to co-exposure treatments, indicating that greater oxidative stress was caused by the mixture of SER and DPH. The induction of AChE activity was increased by the addition of DPH, raising concern about more severe neurotoxicity to fish. More importantly, swimming activity and shoaling behavior were significantly changed by SER alone and its mixture with DPH. In addition, the co-exposure treatments evoked great inhibition on the feeding rate of fish relative to SER alone, which may have more considerable effects at the ecosystem level. To broaden our knowledge of the interactive effects on aquatic organisms, further work on a wider range of psychiatric pharmaceutical mixtures is required.

## Figures and Tables

**Table 1:** Physicochemical properties of sertraline (SER) and diphenhydramine (DPH).

Compound	CAS Number	Formula	Molecular Weight	Water Solubility (mg/L)	Log *p* ^1^	p*K*_a_	Structure
SER	79617-96-2	C_17_H_17_Cl_2_N	306.23	0.15	5.15	9.85	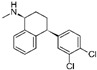
DPH	58-73-1	C_17_H_21_NO	255.35	7.52	3.65	8.87	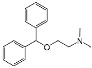

^1^*p* refers to the n-octanol–water partition coefficient.

**Table 2:** The nominal and measured concentrations of SER and DPH in exposure solutions.

Nominal Concentrations (μg·L^−1^)	Actual Exposure Concentration (μg·L^−1^)	
	SER	DPH
Control	ND ^1^	ND
SER (5)	4.17 ± 0.36	ND
SER (5) + DPH (1)	4.34 ± 0.41	0.86 ± 0.14
SER (5) + DPH (5)	4.12 ± 0.33	4.19 ± 0.54
SER (5) + DPH (25)	4.29 ± 0.46	23.5 ± 2.1
SER (5) + DPH (125)	4.19 ± 0.28	111 ± 5.7

^1^ ND: not detected.

## Supplementary Materials

The following are available online at https://www.mdpi.com/1660-4601/16/17/3137/s1, Figure S1: The glass tank used for the tests of swimming activity and shoaling, Figure S2: The glass tank used for the test of feeding rate, Table S1: Experimental conditions used for electrospray tandem mass spectrometry.
