# Supplementary material for: Interactive Effects of Sertraline and Diphenhydramine on Biochemical and Behavioral Responses in Crucian Carp (Carassius auratus)
Source: Int J Environ Res Public Health. 2019 Aug 28;16(17):3137. doi: 10.3390/ijerph16173137 (PMC6747225; doi:10.3390/ijerph16173137)
Supplement: Supplementary file 1 [file ijerph-16-03137-s001.pdf]

Supplementary materials for

Interactive Effects of Sertraline and Diphenhydramine on Biochemical and Behavioral Responses in Crucian Carp (*Carassius auratus*)

Zhengxin Xie [1] and Guanghua Lu[2,3]*

[1] School of Resources and Environment, Anhui Agricultural University, Hefei, 230036, China; xiezx@ahau.edu.com

[2] Water Conservancy Project & Civil Engineering College, Tibet Agriculture & Animal Husbandry University, Linzhi, 860000, PR China; ghlu@hhu.edu.cn

[3] College of Environment, Hohai University, Nanjing 210098, China; ghlu@hhu.edu.cn

Correspondence: ghlu@hhu.edu.cn (G.L.)

1. Sample extraction and chemical analysis

Before extraction with Oasis HLB cartridges (Waters, Milford, USA), water samples were filtered through 0.45 μm glass fiber filters to remove particles. The cartridges were preconditioned by passing 5 mL methanol and 5 mL Milli-Q water. After loading the samples, cartridges were washed with Milli-Q water and vacuum-dried for 30 min. The target analytes were then eluted with 6 mL methanol, and the extracts were concentrated to 1 mL with a gentle stream of nitrogen. The final extract was stored in a 2 mL amber glass vial at -20°C for further chemical analysis.

The samples of 0.25 g for liver and 0.5 g for gills, brain and muscle were weighed and put into a 22 mL stainless steel extraction cell, which was positioned in the system of ASE 350 connected to a three-bottle solvent controller. The extracting solvent was acetone/methanol (1/1, v/v). The operating conditions were as follows: extraction temperature, 70°C; extraction pressure, 1500 psi; preheating period, 5 min; static extraction, 5 min; final extraction volume, 60 mL, flush volume 60% of the cell volume; nitrogen purge, 90 s; and number of extraction cycles, 3. After extraction, a total of 35 mL extract was concentrated to 1-2 mL with an RE-2000 rotary evaporator (Yarong, Shanghai, China) and then diluted with 200 mL Milli-Q water. The dilution was loaded onto a preconditioned Oasis HLB cartridge, as described above for further cleanup.

Chromatographic separation was performed with an Agilent 1290 ultra-high performance liquid chromatograph (UPLC) using an Eclipse Plus C18 column (150 mm × 4.6 mm, 5 μm particle size, Agilent Technologies). The column was maintained at 30°C during the sample analysis. Sertraline (SER) and diphenhydramine (DPH) in tissues were analyzed via a gradient elution method. The mobile phase consisted of eluent A (ultrapure water with 0.1% formic acid) and eluent B (HPLC grade acetonitrile). The gradient started with 20% B for the first 0.5 min, increasing to 90% B in 4.0 min. The 90% B mobile phase was then held for 1 min before decreasing to 20% B over 0.5 min, thereby returning to initial chromatographic condition within 5.5 min. Flow rate was kept at 0.3 mL min^{-1}, and the injection volume was 5 μL.

Mass spectrometric analyses were performed on an Agilent 6460 triple quadrupole mass spectrometer equipped with an electrospray ionization (ESI) source operated in positive mode. The capillary voltage was held at 4 kV and the gas flow was set at 6 L min^{-1}. The pressure of the nebulizer was set at 35 psi. The temperature and flow rate of the sheath gas were 350°C and 9 L min^{-1}, respectively. Multiple reaction monitoring (MRM) mode was used to identify and quantify SER and DPH. Experimental conditions used for electrospray tandem mass spectrometry are shown in Table S1.

Recoveries of target compounds were determined with samples of water, liver, brain, gills and muscle. For each matrix, a sample was spiked for SER and DPH. A duplicate unspiked sample was also prepared, and the recoveries were calculated as the percentages of the measured concentrations in the spiked samples relative to the unspiked samples. Samples and matrix spike recovery tests were performed in triplicate, and the RSDs were less than 20%, indicating a high degree of reproducibility. Matrix recoveries of SER and DPH ranged from 85 to 97% for water, 76 to 104% for liver, 77 to 95% for brain, 87 to 112% for gills and 84 to 106% for muscle. Limits of detection (LODs) and limits of quantification (LOQs) were calculated with signal/noise ratios (S/N) of 3 and 10, respectively. For water samples, the LOD and LOQ of SER and DPH were 0.08-0.11 and 0.30-0.37 ng mL^{-1}, respectively. The LODs and LOQs of DPH from

fish tissues were 0.12–0.31 and 0.44–1.03 ng g^{-1}, respectively. The concentrations of SER and DPH in different fish tissues were reported as wet weight.

2. Behavioral tests

After 7 d of exposure, swimming activity, shoaling, and feeding rate were measured. Swimming activity and shoaling trials were conducted in a glass tank (30 cm high × 30 cm wide × 60 cm long). The tank was divided into two small compartments (15 cm wide) and one large central compartment (30 cm wide) using two transparent glass partitions. In addition, a parallel and a vertical line to partitions was drawn on the bottom to divide the central compartment into four equal segments (Fig. S1). Four similar-sized fish were introduced into one of the smaller compartments as a shoal. The test was started by introducing an individual fish to the center of the large compartment. The test fish was acclimated for 5 min, followed by a 10-min video recording of its movements from above. Swimming activity was assessed by the number of both parallel and vertical lines crossed in in 10 min. To obtain a quantitative measure of shoaling, the number of times the fish crossed the parallel line and the duration of time it spent opposite to the shoal were recorded.

The foraging trial was conducted in a glass tank (30 cm high × 30 cm wide × 50 cm long) containing an incomplete vertical glass partition placed on the right side, leaving a narrow space for introducing the fish (Fig. S2). Ten midge larvae with similar size (10–15 mm) were placed in the bottom on the left side before the fish was placed in the test box. The recording process started when the fish passed the vertical partition and moved toward the midge larvae. If the fish did not move toward the midge larvae within 5 min of starting the recording, the glass partition was removed. The test was digitally recorded with a video camera for 10 min. Feeding rate was quantified as the latency for fish to capture the first, 5th, and last (10th) midge larvae.

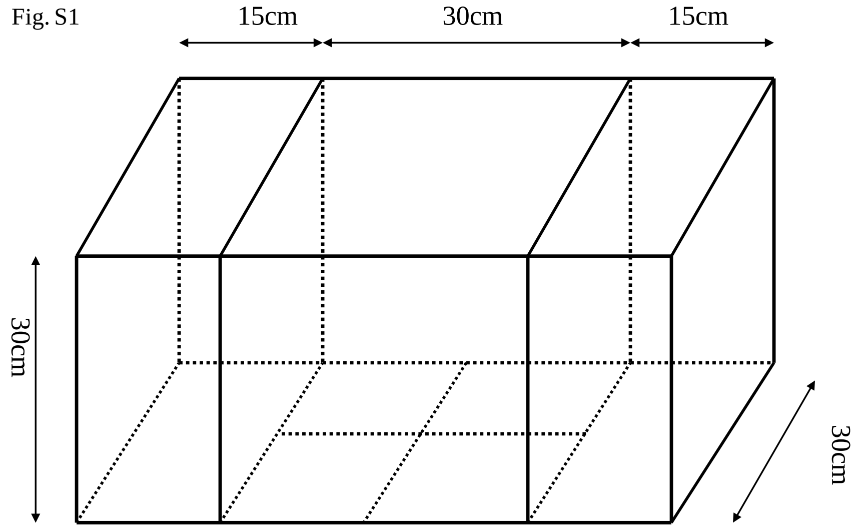

Figure S1. The glass tank used for the tests of swimming activity and shoaling.

Fig. S2

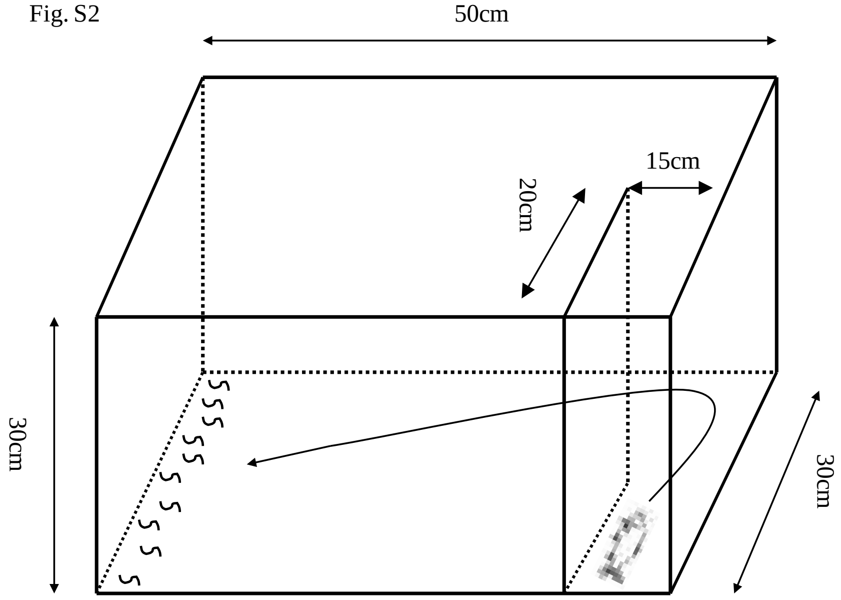

Figure S2. The glass tank used for the test of feeding rate.

Table S1. Experimental conditions used for electrospray tandem mass spectrometry.

Compound	Collision energy (eV)	Fragmentor voltage (V)	Precursor (m/z)	Product ions(m/z)	Modes
SER	20	140	306	159	ESI+
DPH	10	70	256	167	ESI+
